# Arthrofibrosis in Robotic Total Knee Arthroplasty: An Investigation Into How Robotic Assistance May Contribute to a Tight Knee

**DOI:** 10.5435/JAAOSGlobal-D-23-00025

**Published:** 2023-05-02

**Authors:** Muzaffar Ali, Christopher Ferguson, Inderpreet Singh, David Phillips, Shaan Sadhwani, Michael Kahan, Anthony O. Kamson, Nathan Angerett, Richard H. Hallock, Raymond Dahl, Scott G. King

**Affiliations:** From UPMC Pinnacle, Harrisburg, PA (Dr. Ali, Dr. Ferguson, Dr. Phillips, Dr. Singh, Dr. Sadhwani, Dr. Kahan, Dr. Kamson, Dr. Angerett, Dr. Hallock, Dr. Dahl, and Dr. King); the Rubin Institute for Advanced Orthopedics, Baltimore, MD (Dr. Kahan and Dr. Angerett); the University of Maryland Medical Center, Baltimore, MD (Dr. Kahan and Dr. Angerett); the Orthopedic Institute of Pennsylvania, Camp Hill, PA (Dr. Hallock and Dr. Dahl); and the UPMC Arlington Orthopedics, Harrisburg, PA (Dr. King).

## Abstract

Arthrofibrosis after total knee arthroplasty (TKA) can lead to pain and restricted range of motion. Matching native knee kinematics is crucial in avoiding arthrofibrosis postoperatively. However, manual jig-based instruments have demonstrated variability and inaccuracy during primary TKA. Robotic-arm-assisted surgery was developed to increase the precision and accuracy of bone cuts and component alignment. In the literature, limited information on arthrofibrosis after robotic-assisted TKA (RATKA) exists. The purpose of this study was to compare the incidence of arthrofibrosis after manual TKA (mTKA) with RATKA by examining the need for manipulation under anesthesia (MUA) postoperatively and evaluating preoperative and postoperative radiographic parameters.

**Methods:**

A retrospective analysis of patients who underwent primary TKA from 2019 to 2021 was conducted. Rates of MUA were evaluated and perioperative radiographs were analyzed to determine posterior condylar offset ratio, Insall-Salvati Index, and posterior tibial slope (PTS) in patients who underwent mTKA versus RATKA. Range of motion was recorded for patients who required MUA.

**Results:**

A total of 1234 patients were included, of which 644 underwent mTKA, and 590 underwent RATKA. Thirty-seven RATKA patients compared with 12 mTKA patients required MUA postoperatively (*P* < 0.0001). A significant decrease in PTS postoperatively was seen in the RATKA (7.10° ± 2.4° preoperatively versus 2.46° ± 1.2° postoperatively), with a mean decrease of the tibial slope of −4.6° ± 2.5° (*P* < 0.0001). In patients requiring MUA, a larger decrease was seen in the RATKA group when compared with the mTKA group (mean −5.5 ± 2.0 versus −5.3 ± 0.78, *P* = 0.6585). No significant difference was seen in the posterior condylar offset ratio and Insall-Salvati Index in both groups.

**Discussion:**

When conducting RATKA, it is important to match PTS close to the native tibial slope to decrease the incidence of arthrofibrosis postoperatively, as a decrease in PTS can lead to decreased postoperative knee flexion and poor functional outcomes.

Arthrofibrosis, a potential complication after total knee arthroplasty (TKA), is characterized by the production of excessive fibrous scar tissue that can cause significant knee pain and lead to a restricted range of motion (ROM).^1^ Several studies have demonstrated that arthrofibrosis after TKA may worsen postoperative clinical outcomes.^1^ Arthrofibrosis can be a complication that requires a subsequent procedure within the first 5 years postoperatively.^2,3^ As the number of annual TKAs conducted is expected to increase to 3.5 million by 2030, these numbers can be expected to increase accordingly.^4^ To improve clinical outcomes and to decrease the burden on the healthcare system, we should focus on reducing the rates of this prevalent complication.^5^ The causes of arthrofibrosis are multifactorial and can be secondary to surgical techniques such as improper soft-tissue balancing, component malpositioning, inadequate restoration of the posterior condylar offset ratio (PCOR), posterior cruciate ligament tension, and posterior tibial slope (PTS).^6^ Some patient-specific risk factors for developing arthrofibrosis include decreased preoperative knee ROM, smoking, genetics, and diabetes mellitus.^7^ Regarding surgical risk factors, carefully planned bone resections and maintenance of native tibial slope may increase ROM before impingement and substantially improve knee flexion.^8,9^ Although surgeons have generally accepted a target of 3° to 5° of PTS, there has been variability reported in the literature for native tibial slope.^10^ Decreasing tibial slope can lead to a tight flexion gap, which would cause limited knee ROM and the development of arthrofibrosis.^11^ Therefore, approximating the native tibial slope seems appropriate to maintain the natural tension of soft tissues and facilitate natural knee motion and kinematics.^11^ Postoperative factors contributing to arthrofibrosis include poor patient motivation and immobility, delay in starting a rehabilitation program, lack of compliance with prescribed rehabilitation, poor pain tolerance, and infection.^12,13^

Robotic-assisted surgery was developed to increase the precision and accuracy of bone cuts and component alignment in hope of improving functional outcomes and patient satisfaction.^14^ Recent literature suggests that robotic-assisted TKA (RATKA) leads to superior early postoperative outcomes when compared with patients operated on with conventional methods.^15,16^ It is well known that arthrofibrosis is a potential complication after TKA. Arthrofibrosis is estimated to be responsible for 28% of 90-day hospital readmissions and 10% of revision surgeries within the first 5 years postoperatively.^3,4^ However, there is very limited information on arthrofibrosis after RATKA.

The purpose of this study was to evaluate rates of manipulation under anesthesia (MUA) between a consecutive series of patients who underwent RATKA versus a series of patients who underwent TKA with manual instrumentation. Secondarily, we also conducted a radiographic assessment of the PCOR, the Insall-Salvati Index (ISI), and PTS to investigate whether RATKA systematically decreases tibial slope and leads to tight flexion gaps and its effect on the incidence of arthrofibrosis and need for MUA. We hypothesize that RATKA would lead to lower rates of arthrofibrosis after TKA due to the increased accuracy of bony resection, improved implant positioning, and ability to objectively assess the joint line and PTS.

## Methods

### Study Design, Patient Selection, and Demographics

This was a retrospective study with 1234 patients who underwent a unilateral TKA from 2019 to 2021. Of the 1234 patients, 644 patients had manual TKA (mTKA), and 590 patients underwent RATKA with Stryker Mako robotic assistance (Stryker Orthopaedics). Procedures were done by three orthopaedic surgeons at a three-hospital community institution. Stryker triathlon cruciate-retaining implants and insert were used in all patients. The electronic medical record was used to obtain surgical information about the procedures and to determine whether MUA was conducted. Preoperative and postoperative radiological parameters such as PCOR, the ISI, and PTS were measured by two independent surgeons and compared between the two groups. All patients were evaluated a minimum of one year after the index procedure. All patients followed similar postoperative rehabilitation protocols starting on postoperative day zero. There were no specific ROM criteria that were used to determine whether a patient would undergo MUA. Instead, the choice to proceed with MUA was a shared decision-making process between the patient and the surgeon based on the patient's functional status, perceived stiffness, and ROM. Patients who had <90° of flexion before 3 months postoperatively were more often considered for MUA.

Preoperative and postoperative ROM values were obtained by a physical therapist using a goniometer and documented by the physician in the patient's preoperative office visit notes and on follow-up visit notes for all patients who required MUA. No ROM values were collected for patients who did not require an MUA after TKA, as those patients and surgeons were satisfied with their final postoperative ROM, and there was no instability assessed during their postoperative follow-up visit. In addition, these patients were not limited in their activities of daily living.

Institutional review board approval was obtained before conducting the study. Patients were excluded from the study if they underwent aseptic revision surgery after their index procedure, had unresurfaced patella, or had suboptimal radiographs for measurements, extensor mechanism reconstruction, or an underlying disease that promotes excessive scar tissue formation. It is worth noting that exclusion criteria for our study included unresurfaced patella. Any patients who had unresurfaced patella were those who had previously undergone primary TKA at an outside institution and underwent revision TKA for aseptic loosening at our institution. Revision for aseptic loosening was also an exclusion criterion.

### Robotic-Assisted Total Knee Arthroplasty

A standard medial parapatellar approach was conducted in all cases, Stryker implants were used and implanted with assistance from the Stryker Mako system (Stryker Orthopaedics). Before surgery, the Mako (MAKOplasty) TKA application enables the user to do preoperative implant planning using a patient-specific CT-based bone model and virtual implant templates. The prosthesis was digitally aligned using patient bone landmarks as registration points after the attachment of arrays. To achieve a balanced extension and flexion gap, the Mako system allows for the manipulation of femoral rotation, anteriorization, distalization, tibial resection depth, and tibial cut angle to produce well-balanced flexion/extension gaps without the need for ligamentous releases. In our series, the default tibial slope of 2° to 3° was not adjusted. Bony cuts (distal femur, anterior and posterior chamfer cuts, anterior condylar cuts, and proximal tibia) were made using the robotic arm (90° saw and straight saw). The intraoperative target for flexion and extension gaps was 18 to 21 mm depending on surgeon preference and patient specific characteristics including preoperative ROM, flexion contracture, and varus/valgus deformity. This was based on the minimum implant thickness of 17.9 mm referenced in the Stryker MAKO manual which includes the thickness of the distal femur (8.5 mm), the tibial baseplate (3.2 mm), and of a 9 mm polyethylene insert (6.2 mm). Trial components were placed to assess the balance of the knee using MAKOplasty software in extension and 90° of flexion making sure the flexion and extension gaps match and manually assessing the patellar tracking. Cruciate-retaining implants and a polyethylene insert were used in all the patients, and knee motion was retested before closure.

### Manual Total Knee Arthroplasty

A standard medial parapatellar approach was conducted. Conventional intramedullary femoral jig and a tibial extramedullary guide were used with a measured resection technique to obtain alignment. Bony cuts were made in the same order as in the RATKA approach. The patient's native tibial slope was targeted based on the visualized topography of the tibial surface visualized from the front of the knee and the medial aspect of the knee with a stylus inserted through the cutting guide. Trial components were placed to balance the knee in extension and flexion and to assess patellar tracking. Soft-tissue releases were done as needed. Cruciate-retaining implants were used, and knee motion was retested before closure.

### Radiographic Evaluation

Preoperative and postoperative radiographs were analyzed. Measurements of the PCOR, ISI, and PTS were taken and compared between the two groups given their importance in patients' final achievable joint ROM.

### Posterior Tibial Slope

**Figure 1:**
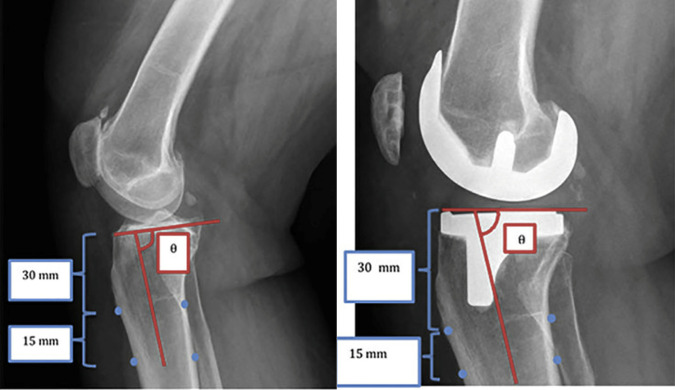
Radiographic determination of PTS in a native knee and TKA. Tibial slope (θ) was determined by the angle formed from a line bisecting the tibial metaphysis and a line tangential to the tibial joint surface on a lateral radiograph (red lines). PTS = posterior tibial slope, TKA = total knee arthroplasty

To assess a patient's PTS, a lateral radiograph of the knee was taken with attention paid to proper overlap of the femoral condyles to ensure appropriate rotational alignment. The preoperative PTS was determined by the angle formed from a line bisecting the tibial metaphysis and a line tangential to the tibial joint surface on a lateral radiograph. This is demonstrated in Figure 1. The same measurements were taken to establish the postoperative tibial slope (Figure 1). These raw measurements were then subtracted from 90 to obtain the true PTS.

### Posterior Condylar Offset Ratio

**Figure 2:**
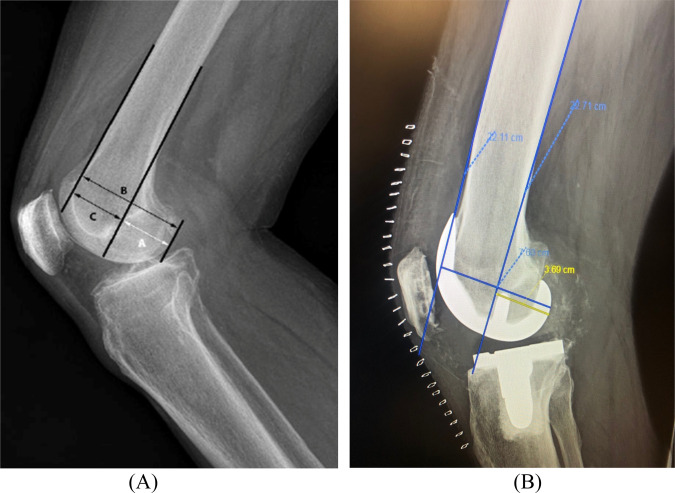
**A,** Radiographic determination of PCOR. PCOR is defined as the ratio of the posterior condyle offset to the diameter of the femur, 2.5 cm above the termination of the supracondylar flare on true lateral view. The first line is drawn tangential to the anterior femoral cortex, and a second line is placed parallel to the first line, tangential to the posterior femoral cortex. A third line is placed parallel to the first two lines, along the posterior aspect of the posterior femoral condyle. “A” represents the PCO, “B” represents the distance from the first line to the third line, and “C” represents the distance from the anterior cortex of the femur to the posterior cortex of the femur. PCOR is defined as A/B. **B,** Radiographic determination of PCOR in a TKA. PCO = posterior condylar offset, PCOR = posterior condylar offset ratio, TKA = total knee arthroplasty

The PCOR is defined as the ratio of the posterior condyle offset to the diameter of the femur, 2.5 cm above the termination of the supracondylar flare on true lateral view (Figure 2). Measurements of PCOR were taken on true lateral preoperative and postoperative radiographs. A line was drawn tangential to the anterior femoral cortex. A second line was placed parallel to the first line, tangential to the posterior femoral cortex. A third line was placed parallel to the first two lines, along the posterior aspect of the posterior femoral condyle. “B” was defined as the distance from the first line to the third line. “A”, which represented the posterior condylar offset (PCO), was defined as the distance from the second line to the third line. PCOR was then defined as A/B.

### Insall-Salvati Index

**Figure 3:**
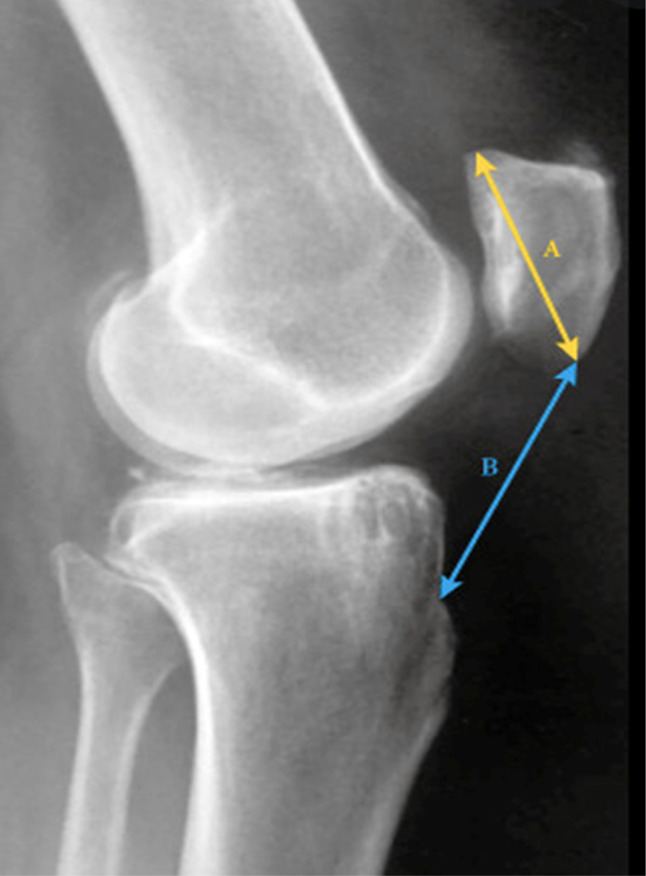
Radiographic determination of ISI. Defined as the PTL over the PL. “A” represents the PL, which is the longest distance from the superior pole of the patella to the inferior most aspect of the patella. “B” represents the PTL, which is the distance from the inferior most aspect of the patella to the superior aspect of the tibial tubercle. ISI = Insall-Salvati Index, PL = patellar length, PTL = patellar tendon length

The ISI was assessed on preoperative and postoperative true lateral radiographs. Patellar length (PL) and patellar tendon length (PTL) were measured. PL was defined as the longest distance from the superior pole of the patella to the inferior most aspect of the patella. PTL was the distance from the inferior most aspect of the patella to the superior aspect of the tibial tubercle. The ISI is defined as PTL/PL (Figure 3).

### Statistical Analysis

We reported continuous variables as mean (range) and categorical variables as number (percent). We used the Student *t*-test to analyze between-group differences for the continuous variables. When it was determined that variances for the comparisons of continuous data were unequal, Welch-Satterthwaite *t*-test statistics were used. We also reported a 95% confidence interval for the mean comparisons. We used the chi-square test to analyze between-group differences for the categorical variables. The Fisher exact test was used when any of the expected frequencies were five or less. All tests were two sided, with the criterion for statistical significance at a *P* value less than 0.05. All the analyses were done by SAS 9.4 (SAS Institute). PTS and ROM values were all reported in degrees. PCOR and ISI were represented as a ratio.

## Results

### Demographics

The patients in the study had a mean age of 65.6 years among both cohorts. The mTKA group had a mean age of 67.5 ± 8.5 years, and the RATKA group had a mean age of 63.8 ± 10 years. There were 253 males (39.2%) in the mTKA group and 260 (44.1%) males in the RATKA group. The average body mass index (BMI) in both groups was 33 kg/m^2^. There was no difference between cohorts in terms of sex or BMI (*P* > 0.05) as seen in Table 1.

**Table 1:** Patient Demographics—Total Sample

	Manual	Robotic	*P* Value
Total no. of patients	644	590	
Patient age Mean (SD), range	67.5 (8.5), 43-90	63.8 (10.0), 27-95	<0.0001
Sex (male), no. (%)	253 (39.29)	260 (44.07)	0.0710
BMI Mean (SD), range	32.9 (6.3), 19.3-56.5	33.1 (6.1), 16-48.4	0.5367
MUA rate, no. (%)	12 (1.86)	37 (6.27)	<0.0001

BMI = body mass index, MUA = manipulation under anesthesia, SD = standard deviation

### Manipulations Under Anesthesia

A total of 49 of the 1234 patients required an MUA postoperatively. There was a statistically significant difference between the cohorts, with 37 (6.27%) RATKA patients versus 12 (1.86%) mTKA patients requiring an MUA (*P* < 0.0001) as seen in Table 2.

**Table 2:** Patient Demographics—MUA Cases

	Manual	Robotic	*P*
Total no. of patients	12	37	
Age, mean (SD), range	65.5 (5.2), 59-75	62.2 (9.4), 42-83	0.1318
Sex (male), no. (%)	9 (75.00)	20 (54.05)	0.3128
BMI, mean (SD), range	33.9 (5.6), 22.8-39.7	32.3 (6.4), 20.5-45.5	0.4321

BMI = body mass index, MUA = manipulation under anesthesia, SD = standard deviation

### Range of Motion in Patients Requiring Manipulation Under Anesthesia

ROM was only recorded for patients who required MUA. There was no statistical difference in knee ROM before TKA in both mTKA and RATKA cohorts. The mean preoperative flexion was noted to be 125° ± 6.4° in the mTKA group and 123.9° ± 5.7° in the RATKA group. Postoperatively, the flexion was significantly reduced to 88.8° ± 5.3° in the mTKA group versus 87.5° ± 5.6° in the RATKA group as seen in Table 3. Postoperatively, there was a mean decrease in 36.3° ± 4.0° of knee flexion in the mTKA group and 36.4° ± 6.1° in the RATKA group as seen in Tables 3 and 4.

**Table 3:** MUA Cases Manual Versus Robotic Tibial Slope, PCOR, ISI, and ROM Findings

	Manual			Robotic			
**Tibial Slope**	**Mean (SD)**	**Range**	**95% CI**	**Mean (SD)**	**Range**	**95% CI**	***P* Value**
Pre-Op	9.25 (1.7)	6-11	8.16-10.34	7.65 (2.2)	4-13	6.92-8.38	0.0253
Post-Op	3.92 (1.7)	1-7	2.85-4.98	2.14 (1.2)	0-5	1.74-2.53	0.0002
Change from Pre-Op to Post-Op	−5.33 (0.78)	−7 to −4	−5.83 to −4.84	−5.5 (2.04)	−11 to −1	−6.20 to −4.83	0.6585
**PCOR**	**Mean (SD)**	**Range**	**95% CI**	**Mean (SD)**	**Range**	**95% CI**	***P* Value**
Pre-Op	0.46 (0.04)	0.41-0.52	0.44-0.49	0.48 (0.04)	0.42-0.54	0.47-0.49	0.1507
Post-Op	0.49 (0.02)	0.46-0.51	0.47-0.50	0.48 (0.04)	0.41-0.57	0.47-0.49	0.3362
Change from Pre-Op to Post-Op	0.02 (0.05)	−0.06 to 0.09	−0.01 to 0.05	−0.004 (0.05)	−0.08 to 0.08	−0.02 to 0.013	0.1152
**ISI**	**Mean (SD)**	**Range**	**95% CI**	**Mean (SD)**	**Range**	**95% CI**	***P* Value**
Pre-Op	0.98 (0.13)	0.83-1.26	0.90-1.06	0.99 (0.11)	0.83-1.26	0.95-1.02	0.8202
Post-Op	1.02 (0.15)	0.75-1.23	0.92-1.11	1.04 (0.10)	0.83-1.26	1.01-1.08	0.5003
Change from Pre-Op to Post-Op	0.04 (0.2)	−0.29 to 0.32	−0.10 to 0.18	0.06 (0.2)	−0.26 to 0.39	0.004-0.11	0.7677
**ROM**	**Mean (SD)**	**Range**	**95% CI**	**Mean (SD)**	**Range**	**95% CI**	***P* Value**
Pre-Op	125 (6.4)	110-132	121-129	123.9 (5.7)	115-135	122-126	0.5845
Post-Op	88.8 (5.3)	79-95	85.4-92.1	87.5 (5.6)	71-95	85.6-89.4	0.5077
Change from Pre-Op to Post-Op	−36.3 (4.0)	−43 to −29	−38.8 to −33.7	−36.4 (6.1)	−49 to −24	−38.5 to −34.3	0.9371

CI = confidence interval, ISI = Insall-Salvati Index, MUA = manipulation under anesthesia, PCOR = posterior condylar offset ratio, ROM = range of motion, SD = standard deviation

**Table 4:** Total Population Manual Versus Robotic Tibial Slope, PCOR, and ISI Findings

	Manual			Robotic			
**Tibial Slope**	**Mean (SD)**	**Range**	**95% CI**	**Mean (SD)**	**Range**	**95% CI**	***P* Value**
Pre-Op	5.89 (1.9)	1-11	5.7-6.0	7.10 (2.4)	2-13	6.9-7.3	<0.0001
Post-Op	6.43 (1.7)	1-11	6.3-6.6	2.46 (1.2)	0-6	2.4-2.6	<0.0001
Change from Pre-Op to Post-Op	0.55 (2.5)	−7 to 7	0.4-0.7	−4.6 (2.5)	−13 to 1	−4.8 to −4.4	<0.0001
**PCOR**	**Mean (SD)**	**Range**	**95% CI**	**Mean (SD)**	**Range**	**95% CI**	***P* Value**
Pre-Op	0.4820 (0.04)	0.41-0.54	0.479-0.485	0.4762 (0.04)	0.40-0.57	0.473-0.480	0.0128
Post-Op	0.4920 (0.03)	0.41-0.61	0.4876-0.4944	0.4810 (0.04)	0.41-0.57	0.4779-0.4842	<0.0001
Change from Pre-Op to Post-Op	0.01 (0.05)	−0.13 to 0.15	0.006-0.0139	0.005 (0.06)	−0.14 to 0.17	0.0002-0.009	0.0924
**ISI**	**Mean (SD)**	**Range**	**95% CI**	**Mean (SD)**	**Range**	**95% CI**	***P* Value**
Pre-Op	0.9989 (0.11)	0.75-1.26	0.990-1.008	1.004 (0.11)	0.78-1.52	0.995-1.014	0.3910
Post-Op	1.01 (0.12)	0.75-1.26	1.002-1.021	1.02 (0.11)	0.75-1.26	1.020-1.011	0.1949
Change from Pre-Op to Post-Op	0.012 (0.17)	−0.43 to 0.42	−0.001 to 0.025	0.016 (0.16)	−0.59 to 0.43	0.002-0.029	0.7222

CI = confidence interval, ISI = Insall-Salvati Index, PCOR = posterior condylar offset ratio, ROM = range of motion, SD = standard deviation

### Posterior Tibial Slope

**Figure 4:**
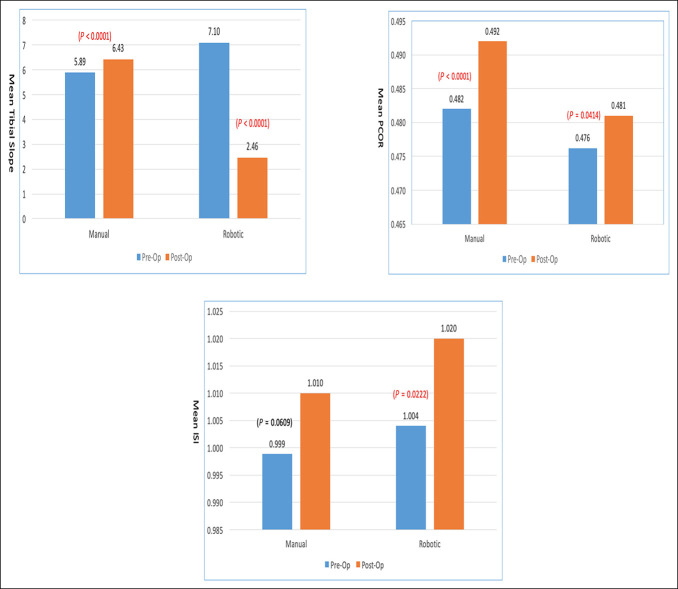
Mean tibial slope, PCOR, and ISI ratio comparison between Pre-Op and Post-Op for manual and robotic TKA. ISI = Insall-Salvati Index, PCOR = posterior condylar offset ratio, TKA = total knee arthroplasty

Overall, PTS was significantly decreased from preoperative measurements for all patients in the RATKA group. The mean PTS in all RATKA patients was 7.10° ± 2.4° preoperatively and 2.46° ± 1.2° postoperatively, with a mean decrease of the tibial slope of −4.6° ± 2.5°. In the mTKA group, the mean PTS in all patients was 5.89° ± 1.9° preoperatively and 6.43° ± 1.7° postoperatively, with a mean increase in the tibial slope of 0.55° ± 2.5° (Figure 4).

**Figure 5:**
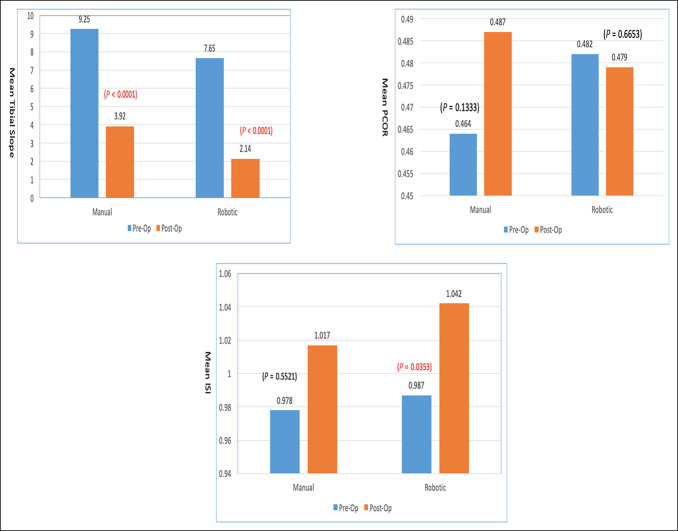
Mean tibial slope, PCOR, and ISI ratio comparison between preoperative and postoperative for MUA manual and robotic TKA. ISI = Insall-Salvati Index, MUA = manipulation under anesthesia, PCOR = posterior condylar offset ratio, TKA = total knee arthroplasty

Looking specifically at patients who required an MUA, there were large decreases in PTS postoperatively in both cohorts. In the RATKA group, the mean preoperative PTS measured 7.65° ± 2.2°, and the mean postoperative PTS measured 2.14° ± 1.2°, with a difference in the mean PTS of −5.5° ± 2.0°. In the mTKA group, the mean preoperative PTS measured 9.25° ± 1.9°, and the mean postoperative measured 3.92° ± 1.7°, with a difference in the mean PTS of −5.3° ± 0.78° (Figure 5). However, there was no statistical difference between the two groups (*P* = 0.65).

### Posterior Condylar Offset Ratio

There was no statistically significant difference observed in PCOR in patients who required manipulation. In those patients, the mean PCOR in the RATKA group was 0.48 ± 0.04 preoperatively and 0.48 ± 0.04 postoperatively with a negligible mean difference of −0.004 ± 0.05 (Figure 4). An insignificant increase in PCOR was noted in patients who required manipulation after mTKA with a mean PCOR of 0.46 ± 0.04 preoperatively and 0.49 ± 0.02 postoperatively (*P* = 0.15) (Figure 5). In general, all the patients in the mTKA and RATKA groups have seen a slight increase in their PCOR postoperatively, which was not statistically significant 0.01 ± 0.05 vs 0.005 ± 0.06, respectively, with a *P* value of 0.092.

### Insall-Salvati Index

No significant difference was observed in the postoperative ISI between cohorts with or without manipulation. The patients who underwent MUA after RATKA had a mean ISI preoperatively of 0.99 ± 0.11 and 1.04 ± 0.10, postoperatively, with a difference of 0.06 ± 0.2 (*P* = 0.76). The ISI in all patients in the RATKA group was 1.00 ± 0.11 preoperatively and 1.02 ± 0.11 postoperatively (Figure 4).

The ISI in patients who required MUA in the mTKA group was 0.98 ± 0.13 preoperatively and 1.02 ± 0.15 postoperatively, with a difference of 0.04 ± 0.2 (Figure 5). The ISI in all patients in the mTKA group was 0.99 ± 0.11 preoperatively and 1.01 ± 0.12 postoperatively.

## Discussion

In the pathogenesis of arthrofibrosis after TKA, fibroblast proliferation and deposition of the extracellular matrix lead to the development of noncompliant scar tissue.^1^ Limited knee flexion can hinder a patient's ability to do daily activities of living and will lead to diffuse pain.^17^ Several factors have been demonstrated to effect postoperative ROM, with the most important being preoperative ROM.^18,19^ In our study, we found no significant difference in preoperative ROM in mTKA patients compared with RATKA patients. Other modifiable factors that play a role in postoperative ROM include implant design and surgical technique, including changes in PCOR, tibial slope, and ISI.^20,21^ We investigated these factors and sought to determine whether the use of a robot for TKA altered these in a meaningful way compared with mTKA.

Preservation of PCOR has been shown to be related to the maximum amount of flexion achieved after TKA.^22^ Bellemans et al^21^ first introduced the concept of PCO and its importance in postoperative ROM after TKA. They determined that an increase in PCO led to ROM limitation due to early impingement between the posterior femoral cortex and the posterior edge of the polyethylene insert. This was in part due to the paradoxical roll forward of the femoral implant during flexion, an elevated posterior lip of the polyethylene component, and a decreased slope of the tibial component.^23^ The smaller the difference between preoperative and postoperative PCOR, the better the ROM one year after surgery.^23^ Massin and Gournay^24^ demonstrated that for each 3-mm decrease in PCO, the knee flexion is reduced by 10°. Subsequent studies confirmed these findings and found that PCO had the most significant effect on postoperative ROM.^23^ Our study showed relative preservation of PCOR in both RATKA and mTKA and demonstrated significantly smaller PCOR difference postoperatively in the RATKA cohort compared with the mTKA. This finding is supported by a study from Sultan et al,^25^ which shows that patients who underwent RATKA had smaller mean differences in PCOR. Therefore, we hypothesize that there are other factors related to the RATKA that can affect the postoperative ROM and necessitate MUA. With RATKA, if the knee is tight in flexion, the option to anteriorize the femoral implant is readily available. Certain mTKA systems also allow you to anteriorize the femoral implant without downsizing. However, most systems require decreasing the size of the femoral implant in anterior referencing systems to remove more bone from the posterior condyles.^26^ Although anteriorizing the femoral implant by small amounts may not significantly affect the average change in PCOR, it may lead to indirect overstuffing of the patellofemoral joint, which can cause a tight extensor mechanism that has been shown to increase anterior knee pain and lead to decreased ROM.^27^

PTS is another important factor related to postoperative ROM. Failure to maintain PTS postoperatively close to the native tibial slope can lead to decreased flexion. Specifically, a decrease in the tibial slope will lead to decreased knee flexion. This is especially true in cruciate-retaining implants.^28,29^ Insall and Kelly^30^ reported that the global PTS was 10° relative to the tibial shaft. It was later shown that slopes of the medial and lateral tibial plateaus are not symmetrical and that these measurements have varied depending on the method of measurement used, presence of osteoarthritis, race, and sex.^31^ The postoperative maximum flexion angle after TKA increases in patients with PTS of 6° or more and is positively correlated with PTS. Several studies have reported that a 1° increase in PTS after TKA improved the postoperative maximum flexion angle by 1.7°.^32^ Schafer et al^33^ demonstrated the use of MAKOplasty software to determine several anatomic measurements. In their study, they found a mean medial tibial slope to be 10.0° ± 6.3° and a lateral tibial slope to be 10.2° ± 3.96°. With further analysis, they found African American patients to have greater medial and lateral tibial slopes than Caucasian patients (10.767 ± 4.493 vs 7.884 ± 3.652 and 10.887 ± 4.475 vs 9.917 ± 3.633, respectively).^33^ Farooq et al^9^ demonstrated the importance of maintaining a PTS close to the native tibial slope. They found that if native tibial slope was changed by more than 5° or if the femoral component was placed in extension or flexion > 10° patients were more likely to be dissatisfied with their total knee arthroplasties.^9^ We found that in patients requiring MUA, there was a significant decrease in the PTS. This was true for both RATKA and mTKA. However, there was a greater decrease in PTS in RATKA compared with mTKA, which correlates with the increased rate of MUA in the RATKA group. The default setting for PTS in the software used for RATKA in this study is 2 or 3°.^34^ There has been extreme variability reported in the literature for native tibial slope, although some studies quote a target of 3° to 5° of a PTS, which complicates using this target on all patients without compromising soft-tissue balance.^10^ In general, approximating the native tibial slope seems appropriate to maintain the natural tension of soft tissues and facilitate natural knee motion and kinematics. This suggests that further research may be warranted to improve our understanding of tibial slope using robotic technology to RATKA outcomes. Our study shows that if the default setting for PTS in RATKAs is routinely used, the PTS will be decreased in most patients. In mTKA, most systems have a default of 3° to 7°, and one can manually add more PTS using the saw freehand. We think that these factors contribute to the differences we observed in PTS and the subsequently increased need for MUA secondary to arthrofibrosis in RATKA patients.

Failure to restore the normal joint line, or causing patella baja, has been reported as a cause of arthrofibrosis. The occurrence of patella baja postoperatively is determined by evaluating for a low ISI. Maeno et al^35^ demonstrated that the development of surgical patella baja after TKA led to the impingement of the patella on the tibial component resulting in restrictive flexion ROM. More commonly in TKA, the joint line is elevated due to overresection of the femur or tibia necessitating the use of a larger polyethylene insert.^36^ This can lead to a decreased ISI, which is referred to as pseudo patella baja and can cause an increase in anterior knee pain and a higher incidence of flexion contracture.^37^ However, we found no difference in change in the ISI in mTKA compared with RATKA. We, therefore, have no evidence to suggest that the increase in postoperative arthrofibrosis and need for MUA seen in RATKA patients is related to an alteration in the joint line. Our finding is supported by Sultan et al,^25^ which shows that the patients who underwent RATKA were less likely to deviate from normal ISI values and therefore less likely to develop patella baja.

There are several limitations to our study. The first is the retrospective design that inherently adds bias regarding causation versus correlation. We also did not obtain the preoperative and postoperative ROM of all patients in both cohorts as those patients and surgeons were satisfied with their final postoperative ROM. In addition, those patients were not limited in their activities of daily living; hence, we focused only on patients who required MUA. This means that it is possible that there is a subset of patients who coped with limited ROM after TKA rather than pursuing MUA. Although this may mean that there were cases of arthrofibrosis unaccounted for, they were not of clinical significance as these patients' functional outcomes were overall satisfactory to the degree that no further treatment was pursued. We were unable to document the preoperative severity of knee deformity, which could contribute to a more difficult surgery and theoretically a decreased ROM postoperatively. Finally, there was also no interobserver variability analysis conducted for the preoperative and postoperative radiographic evaluation of PTS, PCOR, or ISR. Although the RATKA and mTKA cohorts were matched based on age, sex, BMI, and implant design, there are other potentially confounding patient-specific factors that can contribute to postoperative ROM and arthrofibrosis. These include underlying disease, physical activity level, tibiofemoral varus/valgus angle, previous surgeries, patellar diameter, surgical technique, preoperative ROM, postoperative physical therapy regimen, and preoperative pain levels.^38,39^

Our study emphasizes the importance of recognizing that manual and robotic TKA are different procedures with different technical targets. Although more information is available to the surgeon with the software associated with RATKA, it is still the surgeon's responsibility to recognize how to use this information and appropriately apply this to each individual patient and his or her anatomy. There is potential for systematic technical error, which can lead to stiff knees in RATKA when the patient-specific data are not consistently applied to the surgical plan. Thus, we highlight the importance of matching the PTS close to the native tibial slope when conducting RATKA as a decrease in PTS can lead to a decrease in postoperative knee flexion and to poor functional outcomes.^9^ Future studies should focus on evaluating the potential correlation between anteriorizing the femoral implant and resultant overstuffing of the patellofemoral joint. As overstuffing the patellofemoral compartment may contribute to postoperative stiffness, future studies may conduct a subgroup analysis on a cohort of patients with unresurfaced patellae. In addition, randomized controlled trials evaluating changes in PTS and the effect on MUA rates would be valuable to reduce bias and further elucidate this important relationship.
